# Secondary Hematoma Expansion and Perihemorrhagic Edema after Intracerebral Hemorrhage: From Bench Work to Practical Aspects

**DOI:** 10.3389/fneur.2017.00074

**Published:** 2017-04-07

**Authors:** Krista Lim-Hing, Fred Rincon

**Affiliations:** ^1^Department of Neurology, Thomas Jefferson University, Philadelphia, PA, USA; ^2^Department of Neurosurgery, Thomas Jefferson University, Philadelphia, PA, USA

**Keywords:** hypothermia, induced, neurosciences, brain edema, intracranial hypertension

## Abstract

Intracerebral hemorrhages (ICH) represent about 10–15% of all strokes per year in the United States alone. Key variables influencing the long-term outcome after ICH are hematoma size and growth. Although death may occur at the time of the hemorrhage, delayed neurologic deterioration frequently occurs with hematoma growth and neuronal injury of the surrounding tissue. Perihematoma edema has also been implicated as a contributing factor for delayed neurologic deterioration after ICH. Cerebral edema results from both blood–brain barrier disruption and local generation of osmotically active substances. Inflammatory cellular mediators, activation of the complement, by-products of coagulation and hemolysis such as thrombin and fibrin, and hemoglobin enter the brain and induce a local and systemic inflammatory reaction. These complex cascades lead to apoptosis or neuronal injury. By identifying the major modulators of cerebral edema after ICH, a therapeutic target to counter degenerative events may be forthcoming.

## Introduction

Intracerebral hemorrhage (ICH) comprises 10–15% of strokes annually in the United States and associated with the highest morbidity and mortality (1). There is approximately 40% mortality at 1-month postbleed, and only about 20% of those patients regain functional independence (2). A key factor affecting ICH outcome is hematoma size and hematoma expansion. Although death may occur acutely at the sentinel ICH event, delayed neurologic deterioration often occurs with the evolution of the hematoma and injury of the surrounding tissue. Key variables influencing the long-term outcome after ICH are hematoma size and location and are not modifiable at the onset of symptoms. Hematoma growth can also be a predictor of poor outcome, and recent clinical evidence suggests that it may be preventable. Additional risk factors for hematoma growth include antithrombotic therapy, hypertension, large initial hematoma size, and genetic predisposition such as with APOE genotype. Approximately 30% of patients demonstrate significant hematoma expansion during hospitalization (3). Therapies aimed at the prevention of hematoma growth generally target either blood pressure (BP) control or hemostasis. Recent large clinical trials tested therapies aimed at modifying these outcomes and included BP reduction and hemostatic therapy with recombinant factor VIIa (rFVIIa) assisted by computed tomography (CT) angiography (CT-A) findings (4).

Perihematoma edema (PHE) has also been implicated as a contributing factor for delayed neurologic deterioration after ICH (5). Cerebral edema forms within hours in the immediate vicinity of the clot (PHE) and can last for several weeks. PHE is the result of both blood–brain barrier (BBB) disruption and local generation of osmotically active substances that spread to adjacent structures (6). Inflammatory cellular mediators, activation of the complement, by-products of coagulation and hemolysis such as thrombin and fibrin, and hemoglobin spread into the brain tissue and induce a local and systemic inflammatory response. This process likely results from complex chemotactic signals including upregulation of adhesion molecules leading to leukocyte recruitment and migration into the brain tissue (7) (Table 1).

**Table 1:** **Phases of ICH and proposed pathophysiologic events [with permission from Rincon and Mayer (59)]**.

Phase	Event	Time	Implicated mechanism
I	Vascular rupture	1–10 s	Chronic vascular changes: lipohyalinosis, amyloid angiopathy, hypocholesterolemia
II	Hematoma formation	<1 h	Blood pressure, coagulation abnormalities
III	Hematoma expansion	1–6 h	Blood pressure, perihematomal vascular + tissue injury
IV	Edema formation	24–72 h	Cellular and humoral toxicity, blood degradation products

## Hematoma Growth

Studies from ICH models that used histopathology, CT analysis, single-photon emission computed tomography (SPECT), and both conventional CT and CT-A techniques suggested that secondary multifocal bleeding into the perihematoma region is more likely to occur in individuals who experience early hematoma growth. Preliminary histopathological studies provide evidence that the presence of microscopic and macroscopic bleeds in the area surrounding hematoma may represent ruptured arterioles or venules (8). More recent studies that used CT and SPECT techniques have shown that in some patients, early hematoma growth is associated with secondary bleeding in the periphery of the existing hematoma and into congested areas of the perihematoma tissue (9).

Irregular clot morphology, which may represent ongoing or active bleeding after ICH, is another variable that has been associated with (10) early hematoma growth. In support, additional secondary studies from large randomized clinical trials on BP reduction after ICH have also demonstrated that irregular clot morphology is associated with worse long-term outcomes after ICH (11). One study using CT-A technology demonstrated that the presence of active contrast extravasation into the hematoma was associated with subsequent hematoma growth (12) and higher mortality (13) in 30–46% of patients (14, 15). Simultaneous bleeding from multiple lenticulostriate arteries has been demonstrated angiographically immediately after ICH (16, 17). This evidence suggests that early hematoma growth occurs because of bleeding into a congested layer of tissue that forms acutely at the periphery of the hematoma (18). Possible contributing factors include (a) increased local tissue pressure leading to mechanical injury, (b) a local fibrinolytic effect, (c) plasma protease induction, and (d) secondary inflammation related to clotting proteins and end-blood products. However, the relative importance of these factors in the early hours after ICH is unclear (6, 19–21).

A proposed theory is that the increase in local tissue pressure occurs in the brain surrounding the hematoma at the expense of a form of “congestive” tissue ischemia. This phenomenon may be similar to what is seen after cerebral infarction from cortical vein or dural sinus thrombosis. All of these mechanisms coupled with regional mechanical and ischemic tissue damage and the possibility of a local coagulopathic environment may contribute to worsening secondary bleeding from venules and arterioles.

### Clinical and Radiological Evidence in Support of Early Hematoma Growth

Early hematoma growth evidenced by consecutive CT scans can occur in 18–38% of patients scanned within 3 h of ICH onset. Multiple CT-based studies have provided further support for the occurrence of early hematoma growth after ICH (10, 22–26). The highest incidence of early hematoma growth (38%) was seen in the Brott and Broderick’s study, but the investigators concluded that the true frequency of hematoma growth may have been higher because clinical deterioration and immediate surgical intervention precluded the performance of the follow-up CT scans in some of the studied patients (26). To summarize, the only consistently identified predictor of early hematoma growth is the time from the onset of ICH to CT scan, in other words, the earlier the first CT scan is done, the more likely subsequent bleeding will be detected on a follow-up CT scan (10, 25, 27).

To this end, hematoma expansion occurs in only 5% of patients who are initially scanned beyond 6 h of symptom onset (10, 25, 26, 28). Early hematoma expansion is consistently associated with poor clinical outcomes and higher mortality rates versus no expansion. Similarly, significantly greater reductions in the Glasgow Coma Scale and National Institute of Health Stroke Scales have been reported among patients with documented hematoma expansion on 1-h follow-up CT scans versus those without growth (26). These observations suggest that the reduction in hematoma growth may be an important strategy for improvement of survival and outcome after ICH.

## Treatment Strategies to Prevent Hematoma Growth

### BP Control

Patients with ICH should have tightly managed BP, but it is frequently elevated acutely (29). In the majority of cases, extremely high admission BP is the primary therapeutic issue in ICH patients. In the Study of Treatment of Acute Hypertension (30), nearly 30% of patients who presented to an Emergency Department with acute hypertension had a demonstrable brain injury of which 30% were ICHs. Systolic blood pressure (SBP > 140 mmHg) is seen in >75% of patients with ICH (29, 31). Causes of this hypertensive response include upregulation of the sympathetic nervous system and the renin–angiotensin and pituitary-adrenal axis (32). Single-center studies and a systematic review have independently demonstrated a higher risk of early clinical deterioration, mortality, and worst long-term outcome with either extreme high or low levels of BP after ICH (33–38).

Extreme levels of BP could theoretically contribute to acute hematoma growth and later aggravate PHE and intracranial pressure (ICP). This could potentially translate into worst short- and long-term outcomes after ICH (39, 40). Preliminary studies provided some evidence of early hematoma growth from bleeding into an ischemic penumbra zone surrounding the hematoma (41, 42). However, other studies did not confirm the existence of such ischemic and hypoperfused area in the periphery of the hematoma. In the landmark study by Brott et al. (26), acute hypertension after ICH was not associated with hematoma growth, but the authors suggested that the use of antihypertensive agents may have negatively confounded this association. Similarly, acute hypertension was not associated with hematoma growth in the Recombinant Activated Factor VII ICH Trial (43).

Despite this conflicting evidence, the overall consensus is that extreme levels of BP (either low or high) after ICH should be treated carefully. Controversy exists regarding the optimal threshold for treatment and target level [SBP versus mean arterial pressure (MAP)]. Aggressive BP reduction in the setting of impaired autoregulation may predispose to perihematomal or distant brain tissue ischemia, whereas intact autoregulation might result in reflex vasodilation and increase in cerebral edema resulting in higher ICP (44, 45). In a small pilot study of BP reduction after ICH, 14 patients with supratentorial ICH were randomized to receive either labetalol or nicardipine within 22 h of ictus with the aim to lower the MAP by 15%. Cerebral blood flow (CBF) studies were performed before and after treatment with positron emission tomography and [15O] water. No changes in global or perihematoma CBF were observed (46). Finally, earlier studies also demonstrated that a controlled pharmacologically based reduction in BP had no adverse effects on CBF in both humans and animals (47, 48). These preliminary evidence led to the development of clinical studies on aggressive BP control after ICH.

Seven clinical trials have evaluated the role of intensive BP reduction after ICH (49–55). The Intensive Blood Pressure Reduction in Acute Cerebral Hemorrhage Trial (INTERACT)-I phase II study was an open-label trial of 403 patients randomized to a target SBP of <180 (guideline recommendation) or <140 mmHg within 6 h of onset (56). The study showed a trend toward lower relative and absolute hematoma growth from baseline to 24 h in the intensive treatment group (SBP < 140 mmHg) compared with the control group. The phase III clinical trial INTERACT-II concluded that aggressive BP control did not result in a significant reduction in the mortality rate or severe disability after ICH (54, 57). However, a trend was observed when the primary outcome was analyzed in an ordinal fashion, suggesting that in a selected cohort of ICH patients, intensive lowering of BP may improve long-term outcomes (57).

The Antihypertensive Treatment in Acute Cerebral Hemorrhage (ATACH)-I trial (52, 58) confirmed the feasibility and safety of early rapid BP reduction in ICH. This phase II randomized prospective controlled study employed a dose escalation regimen of intravenous nicardipine for BP reduction in 80 patients with ICH. No effect was seen on outcome or neurological worsening. Both INTERACT-II and ATACH-I showed that although early and intensive BP lowering is clinically feasible and safe, this was not associated with meaningful clinical outcome differences (see Table 2).

**Table 2:** **Comparison of INTERACT-II versus ATACH-II trials**.

	Randomization	Treatment group	Control group	Outcome
INTERACT-II	All within 6 h of symptom onset; 48% systolic blood pressure (SBP) > 180 mmHg	Target SBP < 180 mmHg within 1 h after randomization	Target SBP < 140 mmHg with agents of physician’s choosing	No significant reduction in modified Rankin Scale (mRS) score at 3 months
ATACH-II	All within 4.5 h of symptom onset; SBP > 180 mmHg	Maintain SBP 140–179 mmHg during 24 h after randomization	Maintain SBP 110–139 mmHg during 24 h after randomization with first-line nicardipine and second-line labetalol	No significant reduction in mRS score at 3 months

The recently completed phase III ATACH-II clinical study was closed based on futility translating into no meaningful benefit from the intensive treatment group (SBP < 140 mmHg) compared to the guideline recommendation (SBP < 180 mmHg) (53). The ATACH-II trial was designed to evaluate the efficacy of aggressively lowering the SBP in ICH patients but in an earlier time-window (60). One aim of the ATACH-II trial was to show that a more rapid intensive reduction in the SBP level than that used in INTERACT-II would make it more likely to show a larger therapeutic benefit. However, the intensive and early treatment did not result in a lower rate of mortality or poor outcome.

In the Intracerebral Hemorrhage Acutely Decreasing Arterial Pressure Trial (ICH-ADAPT) I (55), the use of a strict triple-regimen BP lowering protocol permitted a significant BP difference between the groups at 2 h postrandomization. The target BP was achieved in 79% of patients in the <150 mmHg group at 2 h. The follow-up phase II ICH-ADAPT II is designed to test the hypothesis that aggressive antihypertensive therapy will alter the natural history of hematoma growth, improving outcomes after ICH using MRI and DWI as primary outcomes. The study will identify biomarkers that may be putative mediators of ischemic injury in ICH patients (49) (see Table 3).

**Table 3:** **Prospective clinical trials of blood pressure management in ICH**.

Trial	Study design	SBP targets	Onset to randomization time	Medical intervention used	End point
INTERACT	*n* = 404, RCT, PROBE, phase II	Standard: <180 Intensive: <140	<6 h	Variable	24 h hematoma growth

INTERACT-II	*n* = 2,839, RCT, phase III	Standard: <180 Intensive: <140	<6 h	Variable	mRs at 90 days

ATACH	*n* = 60, RCT, phase II	Tier 1: <170–200 Tier 2: <140–170 Tier 3: <110–140	<6 h	Nicardipine	Treatment feasibility and safety

ATACH-II	*n* = 1,200, RCT, PROBE, phase III	Standard: <180 Intensive: <140	<4.5 h	Nicardipine	mRS at 90 days

ICH ADAPT	*n* = 75, RCT, PROBE, phase II	Standard: <180 Intensive: <150	<24 h	Labetalol Hydralazine Enalapril	Perihematoma CBF

ICH ADAPT II	RCT, PROBE, phase II	Standard: <180 Intensive: <140	<6 h	Labetalol Hydralazine Enalapril	DWI lesion frequency at 24 h

*RCT, randomized control trial; PROBE, prospective randomized open blinded end point; CBF, cerebral blood flow; mRS, modified Rankin Scale; SBP, systolic blood pressure*.

Current recommendations for Guidelines for the Management of Spontaneous Intracerebral Hemorrhage from AHA/ASA state that for “ICH patients presenting with SBP between 150 and 220 mmHg and without contraindication to acute BP treatment, acute lowering of SBP to 140 mmHg is safe (Class I; Level of Evidence A) and can be effective for improving functional outcome (Class IIA; Level of Evidence B)” (61). This recommendation is consistent with the results of the ATACH-II trial in which participants with intracerebral hemorrhage volume <60 cm^3^ and participants with intracerebral hemorrhage volume <60 cm^3^ and GCS > 5 were assigned to a SBP goal <140 mmHg or SBP < 180 mmHg. The ATACH-II trial did not result in a lower rate of death or disability with acute reduction of SBP to goal <140 mmHg than the standard goal SBP < 180 mmHg. The absolute difference between the two groups in the rate of death or disability was 1 percentage point (60). Although feasible and safe, the rate of renal adverse events within 7 days after randomization in ATACH-II was significantly higher in the intensive treatment group than in the standard-treatment group (9.0 versus 4.0%, *P* = 0.002) (60).

### Hemostasis

As hematoma growth is a powerful predictor of outcome after spontaneous ICH, it makes biological sense to attempt to optimize hemostasis as early as possible. rFVIIa (Novoseven^®^, Novo Nordisk) has been approved for the management of bleeding patients with congenital forms of hemophilia and who are resistant to conventional factor VIII replacement therapy. There is substantial evidence that rFVIIa may optimize hemostasis in patients with normal coagulation function. Recently, a randomized controlled phase II study of 399 patients with spontaneous ICH demonstrated that the administration of rFVIIa at doses of 40, 80, or 160 µg/kg within 4 h of onset was associated with a 38% reduction in death and improved functional outcomes at 90 days, despite a 5% increase in the frequency of arterial thromboembolic adverse events (62). However, this effect was not replicated in the follow-up phase III FAST clinical trial of rFVIIa after ICH. In this study, doses of 80 and 20 µg/kg of rFVIIa were compared against placebo in 841 subjects with spontaneous ICH. The study found no significant difference in the proportion of patients with death or severe disability (mRS 5–6) at 90 days, but the hemostatic effect and adverse effect profiles were replicated (63). On the basis of these results, the routine use of rFVIIa as a hemostatic therapy for patients with spontaneous ICH cannot be recommended. A *post hoc* study of the FAST clinical trial demonstrated that factor rFVIIa may be useful in younger patients who present within an earlier time-window, but further recommendations may need to be supported by future clinical trials (64).

A preliminary clinical study of the antifibrinolytic agent epsilon aminocaproic acid (ECA) was conducted with negative results (65). The management of ICH with Aminocaproic acid open-label pilot study (MANICHAN-PILOT) and the Antifibrinolytic Therapy in Acute Intracerebral Hemorrhage clinical trial are also designed to test the hypothesis that ECA administration within 3 h of ICH is associated with less hematoma growth and improved outcomes (66, 67). As antifibrinolytic therapies carry a higher risk of adverse thromboembolic events, additional studies have focused on determining the specific population of patients that might benefit from this therapy. Four ongoing clinical trials are studying if image-assisted antifibrinolytic therapy may offer further benefit by identifying ICH patients with ongoing bleeding and whom may benefit from acute hemostasis (68–71).

Although CT-A post-ICH is not routinely performed in all clinical centers, it may prove helpful in predicting hematoma growth and clinical outcomes (13, 72). In a prospective study of 39 patients with spontaneous ICH, focal enhancing foci (contrast extravasation, “spot sign”) seen in initial CT-A was associated with the presence and extent of hematoma progression with good sensitivity (91%) and negative predictive value (96%) (14). In the “Spot Sign” Selection of Intracerebral Hemorrhage to Guide Hemostatic Therapy (SPOTLIGHT), ICH patients with a “spot sign” (14) will be randomly assigned to a single injection of rFVII or placebo. The study aims at evaluating the rate of hematoma growth and the difference in proportion of clinical outcomes such as death and disability (69). In the Spot Sign for Predicting and Treating ICH Growth Study (STOP-IT), investigators will determine whether CT-A can predict which individuals with ICH will experience significant hematoma growth in the size of the hemorrhage and the effect of rFVIIa on hematoma growth (68). The end points of the Tranexamic Acid for Acute ICH Growth prEdicted by Spot Sign (TRAIGE) clinical trial and the Spot Sign and Tranexamic Acid On Preventing ICH Growth—AUStralasia Trial (STOP-AUST) are similar to STOP-IT, but in this study, the investigators will use tranexamic acid, a newer antifibrinolytic agent (70, 71).

Additional studies related to hemostasis involve the use of platelet transfusions and prothrombin complex concentrate (PCCs) for coagulopathic or antiplatelet-exposed ICH patients and optimization of coagulation before neurosurgical interventions (73–76). One study recently assessed the effect of platelet transfusion in an open-label inception cohort of ICH patients who underwent platelet function assays with Accumetrics (75). In those patients with abnormal platelet function results and risk of poorer outcome, early platelet transfusion improved platelet activity and was associated with smaller hematoma sizes and with a better functional outcome at 3 months (75). The recently finished Platelet Transfusion in Cerebral Hemorrhage (PATCH) clinical trial (73) aimed at determining whether platelet transfusion improves the risk of hematoma growth and functional outcome in ICH patients who were taking antiplatelet agents. The PATCH study demonstrated significant adverse events, higher mortality, and worst long-term functional outcome in ICH patients who received transfusion. On the basis of the results of this study, platelet transfusion cannot be recommended as a standard procedure in this specific patient population.

Other approaches to optimize antifibrinolytic therapy have been used in combination with surgical evacuation. In the “Intraoperative intravenous administration of rFVIIa and hematoma volume after early surgery for spontaneous intracerebral hemorrhage clinical trial,” the administration of intravenous rFVIIa did not change the hematoma volume or the functional outcome after ICH combined with early surgery. Interestingly, the study showed that there were no meaningful differences in the rates of deep venous thrombosis, myocardial infarction, or cerebral ischemia (77). In the “International Normalized Ratio (INR) Normalization in Coumadin Associated Intracerebral Hemorrhage phase III clinical study,” investigators will test the hypothesis that the treatment for coagulopathic ICH with PCC improves normalization of the INR, hematoma growth, and clinical outcomes compared to transfusions of fresh-frozen plasma (74).

## PHE after ICH

The secondary injury of ICH results is the formation of PHE, which may contribute to an increase in peri-hematoma volume by at least 75% (78). This progression of neuronal injury may lead to increased ICP, herniation, neurological deficits, and death. Enhanced models of hydrostatic and osmotic forces have been recently formulated to explain PHE based on the unique properties of the BBB. Early PHE is attributed to the transcapillary efflux of electrolytes and water from blood vessels (ionic edema), osmotically active serum proteins, and cytotoxic edema from neuronal energy failure. Delayed PHE is produced by the BBB disruption (vasogenic edema) and neuronal death (cytotoxic edema) (20). Three intertwined neurotoxic cascades contribute to the development of delayed PHE: inflammation, erythrocyte lysis, and thrombin production (79). The combination of these processes results in BBB disruption and death of brain parenchyma cells (Table 4).

**Table 4:** **Mechanisms implicated in the genesis and worsening of perihematomal edema**.

Early		Late
<24 h	24–72 h	>72 h
Serum proteins Glucose Electrolytes (Na, K)	Cellular toxicity (white blood cells, platelets) Humoral toxicity [interleukin (IL)-1, IL-6, intracellular adhesion molecule, tumor necrosis factor alpha, prostaglandins, leukotrienes, vascular endothelial growth factor, complement] Coagulation cascade (thrombin, fibrinogen, t-PA) Glutamate and amino acids Epinephrine?	Blood degradation products (Hgb, Fe, biliverdin) Nitric oxide Free radicals Apoptosis Matrix metalloproteinases Glutamate and amino acids

*With permission from Ref. (59)*.

Edema formation after ICH progresses through several phases: a hyperacute phase involves transendothelial osmotic pressure, clot retraction, and cytotoxic edema in the first several hours; an acute phase in the first day involves the clotting cascade, thrombin production, and inflammatory activation; and a third phase, beginning approximately 72 h post-ICH, involves erythrocyte lysis and hemoglobin-induced neurotoxicity (80, 81). In general PHE progresses over 24 h, then remains relatively constant for about 4 days and resolves over a period of several weeks (6). Some studies suggest that a “penumbra” of progressive tissue damage and edema develop in the perihematomal region (82). A 75% median increase in relative edema and 100% median increase in absolute edema volume over the first 24 h after ICH has been observed (83). In one study using SPECT, perilesional CBF normalized from initially depressed levels as PHE formed during the first 72-h post-ICH. The eventual degree of PHE was associated with the volume of reperfused tissue implicating reperfusion injury in the pathogenesis of PHE formation (9). A significant heterogeneity in CBF can occur after ICH with lower CBF (hypoperfusion) near the hematoma and higher CBF (hyperperfusion) in healthy overlying subcortical and cortical regions. The vasodilatory response of pial arteries in the periphery of the injury zone may reflect a local inflammatory reaction.

### Hyperacute Phase

In this phase, PHE starts with the extravasation of serum into the brain parenchyma and cellular dysfunction before BBB disruption. Several early pathophysiologic events leading to early PHE are related to the blood itself, which acts as a “neurotoxic” substance (80, 81). During the first hours after ICH, clot retraction occurs with decreasing clot volumes and increasing PHE volume. Plasma protein extravasation acts oncotically increasing the interstitial osmotic pressure to induce rapid PHE development, extravascular coagulation, and fibrin deposition (80, 81). Experimental evidence strongly supports the hypothesis that hyperacute PHE is largely composed of peripherally exuded serum proteins after clotting of the hematoma and consumption of plasma clotting factors (83–85). Cytotoxic edema secondary to transcellular shifts in Na+ and Cl− has also been shown to increase in the early phases after ICH (85). Within hours, the ensuing cytotoxic edema contributes to the transendothelial trans-BBB osmotic pressure gradient. The mechanisms implicated in early cytotoxic edema involve the extracellular accumulation of neurotoxins such as glutamate, which is associated with further mitochondrial and Na-ATP pump failure (86).

### Acute Phase

Delayed edema formation may result from neuroinflammation and erythrocyte lysis, mediated by the local and systemic upregulation of chemotaxins, the complement system activation, and the release of thrombin and hemoglobin by-products (87). Inflammatory mediators also enhance early PHE after ICH by activation of leukocytes and generation of chemokines and chemotaxins *via* activation of the transcription factor NF-κB (88). Both immunochemical and physical stress in the PHE area lead to NF-κB activation with subsequent pattern recognition by receptors such as toll-like receptors 2 and 4 and thrombin engagement *via* protease-activated receptors (PARs). After activation, the NF-κB leads to upregulation of target genes that encode for cytotoxic cytokines, chemokines, and matrix metalloproteinases (MMPs), which are implicated in further BBB disruption.

White blood cells (WBCs) and neutrophils infiltrate the hemorrhagic brain within 4–5 h and may cause neuronal damage by producing reactive oxygen species and pro-inflammatory proteases (89). Leukocytes die by apoptosis within 2 days after entering the hemorrhagic brain damaging brain tissue through microglia and macrophage activation (79). In this process, glutamate and other excitotoxic amino acids continue to accumulate transiently in the extracellular fluid of the perihematomal region. In one study, peak elevation in glutamate concentrations was observed as early as 30 min post-ICH (90, 91).

Two hypotheses may explain the release of the neurotoxic amino acids: ischemia and cellular trauma. In the setting of ischemic injury, release of amino acids has been recorded and cellular trauma to neurons has also been known to release intracellular stores of glutamate into the extravascular space. Damage to astrocytes normally involved in the removal of glutamate may also exacerbate the accumulation of extracellular glutamate and other excitotoxic amino acids. Extraneuronal glutamate-based neurotoxicity is the result of activation of the postsynaptic *N*-methyl-d-aspartate receptor. The activation of this receptor leads to cellular influx of Ca++ and Na+, leading to transcellular ionic shift, cellular edema by flow of free water, and subsequent neuronal death (92). Previous reports show that even a transient exposure to glutamate can result in enhanced neurotoxicity (93). One study for example showed that high concentrations of glutamate in blood within the first 24 h of symptom onset were associated with an exaggerated subacute pro-inflammatory response and worst clinical outcome and increased volume of the residual cavity after ICH (94). This supports the hypothesis that excitotoxicity and inflammation have a role in PHE formation and secondary neuronal death after ICH.

In addition to glutamate, high concentrations of IL-6, tumor necrosis factor alpha (TNF-a), and intracellular adhesion molecule-1 (ICAM-1) have been detected in the hypodense area surrounding the hematoma. TNF-a was shown to increase BBB permeability and cause WBC activation after its administration in a piglet model of ICH (95). It may also has an effect on other intracellular pathways such as G-protein-coupled activation of phospholipases, which lead to the generation of free radicals and an unhealthy redox state (96). Complex mechanisms in the injured neuronal tissue activate both pro- and anti-inflammatory mediators for at least 7 days after ICH. In parallel, transcript expression of pro-inflammatory cytokines rise rapidly within 6 h after the onset of ICH (97). In addition to these mechanisms, local and systemic inflammatory mediators also enhance tissue damage by the activation of WBCS and generation of prostaglandins and leukotrienes (59).

Although CNS tissues are normally immunologically privileged, BBB disruption may be expected to facilitate immune cell entry into the peri-hemorrhagic tissue. Major inflammatory cells that are activated and accumulate within the brain after ICH are primarily brain trafficked monocytes and resident microglia (7, 98–105). Although pro-inflammatory cytokines IL-1/IL-6/TNF-a can be released by many cell types, including microglia, monocytes, and endothelial cells, their principal sources in the brain are from activated microglia and brain trafficked monocytes (105) The initial inflammatory response after ICH is primarily orchestrated by the cytokines, TNF-a, and IL-1b, which are upregulated within hours and increase BBB permeability and allow entry of peripheral immune cells. The cellular sources of these cytokines change during the 7-day time course (106). When edema forms, activated microglia contributes to the pathologic process through inflammatory cells and mediators and cytokine release. TNF-a is present as early as 1 day after ICH suggesting that synthesis and secretion of cytokines may be an early response of microglia to ICH (7). It has been demonstrated that microglia can also be maximally activated at 7 and 10 days after ICH, which is the time frame when the hematoma is being reabsorbed. The ICAM-1 is inducible by tissue injury and other inflammatory cytokines (107). The induction of ICAM-1 in neurons could promote the attachment of WBCs to neurons inducing neuronal injury through direct cell-to-cell interaction and the release of cytotoxic substances (7).

Neutrophils are also implicated in the development of PHE *via* production of cytotoxic molecules such as pro-inflammatory cytokines, reactive oxygen radical species, and MMPs (108). This early inflammatory response has become an attractive therapeutic target. By inhibiting activation and migration of inflammatory cells, research has shown that minocycline, a tetracycline derivative, may reduce microvessel loss, plasma protein extravasation, and edema in addition to cytokine expression by reducing the upregulation of some pro-inflammatory cytokines such as TNF-a and MMP-12 (109). This substance has been shown to reduce specific cytokines (TNF-a and IL-1b) and MMPs implicated in BBB damage (110).

The complement system is classically excluded from the brain by the BBB, but it could enter in its activated form at the time of ICH or through BBB disruption (111). Complement-mediated brain injury is also assisted by the formation of the membrane attack complex (MAC) and the pro-inflammatory response that follows.

The MAC consists of the complement’s C5b–9 particles assembled after its activation. Once activated, the MAC inserts into the cell membrane forming a pore. The formation of MAC in ICH models induces erythrocyte lysis, which has been implicated in the generation of PHE (87). Not only does MAC cause cell lysis but it also modulates other cellular pathways implicated in the generation and release of cytokine, eicosanoid, and oxygen radical species (112). Similarly, MAC insertion may also occur in neurons and endothelial cells, causing neuronal death, cytotoxic edema, and BBB leakage through damage of endothelial cells. In animal models of ICH, therapeutic blockade of receptors for C3a and C5a resulted in less neutrophilic infiltration and lower brain water content compared with no treatment.

The MMPs are a family of proteolytic enzymes involved in the reorganization of the extracellular matrix. Specifically, MMP-9 and MMP-2 degrade major components of the cellular wall’s basal lamina and can disrupt the BBB (113). MMP-9 has been linked to the pathophysiology of different neurological conditions such as multiple sclerosis and cerebral ischemia (114, 115). One study showed that the levels of MMP-9 increased within the first 24 h in patients with supratentorial ICH and that the concentrations of MMP-9 positively correlated with PHE volumes in deep ICH (113).

Levels of MMPs and plasminogen activators are increased 16–24 h after in experimental collagenase-induced intracerebral hemorrhage models, suggesting that agents that block MMPs may reduce the swelling after ICH (21). In this model, treatment with an MMP inhibitor significantly reduced the brain edema in sites distant from the primary lesion, suggesting that the inhibitor blocked vasogenic edema. Excessive proteolysis is normally prevented by tissue inhibitors of MMPs, but during the inflammatory process, the balance is disturbed as their natural inhibitors (TIMPs) are destroyed, favoring proteolysis. Astrocytes, endothelial cells, and microglia secrete MMPs as inactive zymogens that must be activated by other enzymes such as plasmin and free radicals (21). One study investigated the temporal profile of MMPs and their natural inhibitors after ICH and showed increased MMP-9 with PHE and increased MMP-3 with mortality (5). MMP-3 is laminin whose degradation leads to neuronal death. The main form of cell death associated with ICH in the perihematomal region has been shown to be apoptosis during the first days and necrosis from inflammation after 5 days of symptom onset (90, 91). The apoptotic pathway in ICH may involve nuclear factor kappa beta, which is a transcription factor controlling MMPs (116). TIMPs are now recognized as exerting both MMP inhibition and antiapoptotic properties (5).

Early edema formation is associated with both the activation of the coagulation cascade and the generation of thrombin (80, 81). Thrombin is a serine protease produced in the brain immediately after ICH (117). It activates potentially harmful pathways such as apoptosis, microglia activation, and glutamate potentiation. Thrombin-induced brain edema results partly from the disruption in the BBB and may be mediated by the complement cascade. Thrombin is formed in the clot almost immediately after an ICH, but the influx of prothrombin into the brain tissue due to BBB disruption serves as a secondary source (6). Intracerebral infusion of thrombin showed an increase in complement C9 and deposition on neuronal membranes (118). Thrombin-cleaved C3a-like fragments are chemotactic for WBCs and can induce enzyme release for neutrophils (119). Higher levels of TNF-a were seen after the infusion of thrombin in one animal model of ICH (120).

The most direct effect of thrombin is its role in the coagulation cascade with cleavage of fibrinogen to fibrin and other effects are protein mediated. PARs regulate some of the pathological effects of thrombin and are involved in CNS pathophysiology in some animal studies (121). Thrombin is associated with permeability change in the BBB leading to edema through chemotaxin and MMP activation, as well as the release of vascular endothelial growth factor (VEGF) from neurons through receptor activation (122). VEGF increased vascular permeability and vasodilation *via* nitric oxide induction and may serve as a potential fuel for free radical generation (59). Thrombin at high concentrations also kills neurons and astrocytes *in vitro* (123). It can be detrimental at high concentration and protective at low concentrations (118). To this end, thrombin-mediated brain injury has been identified as a possible therapeutic target after ICH. Administration of a thrombin inhibitor may effectively limit PHE formation and secondary neuronal damage (124). In the GUSTO-1 trial, the onset of symptomatic ICH arising as a complication of thrombolysis for acute myocardial infarction was ascertained and the potential mechanisms studied. The *psot hoc* analysis of GUSTO-1 study showed that there was minimal PHE when the patients received thrombolytic therapy, suggesting that thrombin levels may have been affected (125). Argatroban-mediated inhibition of thrombin proved effective in reducing the degree of PHE in a rodent model of ICH (126). The plasminogen activator inhibitor-1 (PAI-1) protein levels increase in the rat brain after ICH or thrombin infusion but brain levels of tPA remain unchanged (127). The upregulation of PAI-1 after ICH implies that endogenous inhibitors of thrombin may limit brain injury and could be potential future therapeutic agents. Although thrombin formation occurs rapidly after ICH, it may remain within the clot and linked to fibrin, which can in turn lead to delayed release of thrombin and therefore delayed PHE (126).

Hyperglycemia can induce inflammatory reactions in ICH leading to neuronal death (7, 128). Activated blood components triggered by high glucose levels may induce increased inflammatory cytokine activity such TNF-a and IL-1, which exacerbates BBB permeability causing vasogenic edema (129). Hyperglycemia increases the secretion of IL-1b in cultured endothelial cells and TNF-a in epithelial cells (130, 131). Free radical formation is increased with hyperglycemia-induced brain injury leading to increased BBB permeability and brain edema (132). Hyperglycemia also induces bradykinin-mediated vasodilation and inflammation. Bradykinin increases BBB permeability and facilitates extravasation by dilation of arterial blood vessels (133).

### Subacute Phase

Thrombin and the coagulation cascade play a major role in acute edema formation following ICH as shown when thrombin inhibitors markedly reduce edema formation (84). In contrast, injection of whole red blood cells into brain fails to induce edema formation by 24 h (19). This delayed RBC lysis may be attributed to either activation of complement system and formation of MAC (134) or depletion of intracellular energy reserves (135). Hemoglobin has been shown to cause brain injury through inhibition of sodium/potassium ATPase activity, toxic radical generation, and lipid peroxidation (80, 81). In one study, upon injection of packed RBCs, there was no edema formation 24 h postinjection, but marked edema after 3 days. This delayed edema is explained by RBC lysis and hemoglobin release (80, 81). Hemoglobin release from erythrocyte lysis increases during the first few days after an ICH (136). Studies show that PHE formation after thrombin injection peaks at 1–2 days, whereas delayed edema formation from erythrocyte lysis peaks at 3 days (80, 81).

The adverse effects of hemoglobin in models of ICH may be due to the molecule itself or by its breakdown products. Hemoglobin rapidly activates lipid peroxidation directly in the first phase and then *via* iron, one of its breakdown by-products, in the following phase (137). Haptoglobin may inhibit phase I, whereas deferoxamine, an iron chelator, and transferrin, an iron-binding protein, can inhibit phase II. The heme from hemoglobin may be broken down by heme oxygenase (HO) into iron (a potent catalyst for lipid peroxidation), carbon monoxide, and biliverdin (138). In turn, the enzyme biliverdin reductase catalyzes the conversion of biliverdin to bilirubin. Both carbon monoxide and iron can stimulate free radical formation leading to PHE. HO is primarily increased in microglia cells around ICH, which are also the most ferritin- and iron-positive cells (139). The intra-cortical injection of iron, causes focal epileptiform discharges and more brain swelling (140). One study demonstrated that hemoglobin also upregulated HO levels in the brain and that HO inhibition by SnPP molecules reduced the classically observed hemoglobin-induced brain swelling (141). HO overexpression and reactive iron accumulation are also associated with oxygen and free-radical species-related cytotoxicity (142). Early expression of HO may result from induction of other plasma proteins and thrombin as well (143).

Intracerebral iron infusions have been shown to cause brain edema and free radical formation leading to neuronal damage (144). Erythrocyte lysis results in the accumulation of non-heme iron molecules in the brain tissue starting at day 3 and reaching its acme after about 7 days of ICH onset. The temporal relationship between HO and non-heme iron levels indicates that an increase in perihematomal HO levels may modulate home-based degradation and iron overload/toxicity in the brain with ICH (145). Previous studies have shown that hemoglobin is toxic to spinal neurons *via* an iron-dependent, oxidative mechanism involving a hydrogen peroxide intermediate factor (146). Iron reacts with lipid hydroxyperoxides to produce free radicals. Deferoxamine, an iron chelator, is routinely used to treat hemochromatosis caused by iron toxicity. It also has been shown to reduce hemoglobin-induced cerebral edema, indicating that iron is a key factor in the pathway for delayed PHE formation after ICH. The degree of secondary neuronal damage may be limited by changes in iron-handling proteins. Specifically, an upregulation in the brain’s iron-capturing protein ferritin may be neuroprotective.

Reactive oxygen species cause brain injury *via* many different pathways. Deoxyribonucleic acid is vulnerable to oxidative stress and markers have been observed at the perihematomal zone demonstrating that oxidative DNA damage is involved in hemorrhagic brain injury (144). Iron can induce lipid peroxidation and stimulate free radical formation, which in turn may cause DNA damage (140). Although apoptotic DNA damage may occur from erythrocyte-induced brain injury, non-apoptotic DNA damage may play an important role because iron levels in the brain are high after erythrocyte lysis (147). The degree of oxidative damage in tissues is limited by a number of free radical scavenging systems. Intracerebral infusion of lysed RBCs causes marked brain edema associated with increased protein carbonyl content and DNA damage and thus reflecting oxidative stress (147). The oxidative stress may be due to upregulation in HO with resultant iron release.

## Conclusion

Spontaneous forms of ICH account for 10–20% of all strokes annually in the United States and is associated with the highest all-time morbidity and mortality (148). Among the factors related to poor prognosis after ICH are the hematoma size, growth, and PHE. To determine therapeutic targets, it is important to evaluate the mechanisms of each phase. Information of the pathophysiologic mechanisms of injury have been identified as primary and secondary.

Hematoma growth in the setting of ICH has a multifactorial etiology with contributing factors such as increased local tissue pressure, a fibrinolytic effect, plasma protease induction, and secondary inflammation. Reduction in hematoma size and growth will likely be important futuristic strategies to improve survival and outcome after ICH. This depends heavily on both BP management and hemostasis. Seven clinical trials have evaluated the role of intensive BP reduction after ICH and ultimately concluded that intensive lowering of BP (SBP < 140 mmHg) provides no significant improvement in outcome compared to the standard goal of SBP < 180 mmHg. Medical treatment for hemostasis varies if the patient was taking an anticoagulant or had an inherent coagulopathy. However, in spontaneous ICH, not related to anticoagulation, rFVIIa has been evaluated as a means to reduce hematoma growth and PHE formation. Although a phase II randomized clinical trial showed that treatment with rFVIIa reduced hematoma growth and improved clinical outcome after ICH, a conclusive phase III clinical trial failed to demonstrate any significant benefit.

The primary injury of hematoma formation and clot retraction occurs followed by secondary injury with BBB disruption complicated by inflammation, coagulation, thrombin, and erythrocyte lysis. Edema formation after ICH progresses through several phases: a hyperacute phase, an acute phase, and a third phase, beginning approximately 72 h post-ICH. These complex cascades lead to worsening edema through BBB permeability and an end point of apoptosis or neuronal injury. By identifying the major modulators of cerebral edema after ICH, a therapeutic target to counter degenerative events may be forthcoming.

## Author Contributions

KL-H and FR contributed equally to the development of this manuscript.

## Conflict of Interest Statement

The authors declare that the research was conducted in the absence of any commercial or financial relationships that could be construed as a potential conflict of interest.

## References

[1] Rincon F Mayer SA. The epidemiology of intracerebral hemorrhage in the United States from 1979 to 2008. Neurocrit Care (2013) 19(1):95–102. 10.1007/s12028-012-9793-y 23099848

[2] Keep RF Hua Y Xi G. Intracerebral haemorrhage: mechanisms of injury and therapeutic targets. Lancet Neurol (2012) 11:720–31. 10.1016/S1474-4422(12)70104-7 22698888 PMC3884550

[3] Dowlatshahi D Demchuk AM Flaherty ML Ali M Lyden PL Smith EE Defining hematoma expansion in intracerebral hemorrhage: relationship with patient outcomes. Neurology (2011) 76:1238–44. 10.1212/WNL.0b013e3182143317 21346218 PMC3068004

[4] Brouwers HB Greenberg SM. Hematoma expansion following acute intracerebral hemorrhage. Cerebrovascular Dis (2013) 35(3):195–201. 10.1159/000346599 23466430 PMC3743539

[5] Alvarez-Sabín J Delgado P Abilleira S Molina CA Arenillas J Ribó M Temporal profile of matrix metalloproteinases and their inhibitors after spontaneous intracerebral hemorrhage. Stroke (2004) 35:1316–22. 10.1161/01.STR.0000126827.69286.90 15087562

[6] Yang GY Betz AL Chenevert TL Brunberg JA Hoff JT. Experimental intracerebral hemorrhage: relationship between brain edema, blood flow, and blood-brain barrier permeability in rats. J Neurosurg (1994) 81:93–102. 10.3171/jns.1994.81.1.0093 8207532

[7] Gong C Hoff JT Keep RF. Acute inflammatory reaction following experimental intracerebral hemorrhage in rat. Brain Res (2000) 871(1):57–65. 10.1016/S0006-8993(00)02427-6 10882783

[8] Fisher C Pathological observations in hypertensive intracerebral hemorrhage. J Neuropathol Exp Neurol (1971) 30:536–50. 10.1097/00005072-197107000-00015 4105427

[9] Mayer SA Lignelli A Fink ME Kessler DB Thomas CE Swarup R Perilesional blood flow and edema formation in acute intracerebral hemorrhage: a SPECT study. Stroke (1998) 29(9):1791–8. 10.1161/01.STR.29.9.1791 9731596

[10] Fujii Y Tanaka R Takeuchi S Koike T Minakawa T Sasaki O. Hematoma enlargement in spontaneous intracerebral hemorrhage. J Neurosurg (1994) 80(1):51–7. 10.3171/jns.1994.80.1.0051 8271022

[11] Delcourt C Zhang S Arima H Sato S Salman RA Wang X Significance of hematoma shape and density in intracerebral hemorrhage: the intensive blood pressure reduction in acute intracerebral hemorrhage trial study. Stroke (2016) 47(5):1227–32. 10.1161/STROKEAHA.116.012921 27073236

[12] Murai Y Takagi R Ikeda Y Yamamoto Y Teramoto A. Three-dimensional computerized tomography angiography in patients with hyperacute intracerebral hemorrhage. J Neurosurg (1999) 91(3):424–31. 10.3171/jns.1999.91.3.0424 10470817

[13] Becker KJ Baxter AB Bybee HM Tirschwell DL Abouelsaad T Cohen WA. Extravasation of radiographic contrast is an independent predictor of death in primary intracerebral hemorrhage. Stroke (1999) 30(10):2025–32. 10.1161/01.STR.30.10.2025 10512902

[14] Wada R Aviv RI Fox AJ Sahlas DJ Gladstone DJ Tomlinson G CT angiography “spot sign” predicts hematoma expansion in acute intracerebral hemorrhage. Stroke (2007) 38(4):1257–62. 10.1161/01.STR.0000259633.59404.f3 17322083

[15] Rizos T Dorner N Jenetzky E Sykora M Mundiyanapurath S Horstmann S Spot signs in intracerebral hemorrhage: useful for identifying patients at risk for hematoma enlargement? Cerebrovasc Dis (2013) 35(6):582–9. 10.1159/000348851 23859836

[16] Mizukami M Araki G Mihara H Tomita T Fujinaga R Arteriographically visualized extravasation in hypertensive intracerebral hemorrhage. Report of seven cases. Stroke (1972) 3(5):527–37. 10.1161/01.STR.3.5.527 4652727

[17] Komiyama M Yasui T Tamura K Nagata Y Fu Y Yagura H. Simultaneous bleeding from multiple lenticulostriate arteries in hypertensive intracerebral haemorrhage. Neuroradiology (1995) 37(2):129–30. 10.1007/BF00588628 7760998

[18] Takasugi S Ueda S Matsumoto K Chronological changes in spontaneous intracerebral hematoma – an experimental and clinical study. Stroke (1985) 16(4):651–8. 10.1161/01.STR.16.4.651 4024177

[19] Lee KR Colon GP Betz AL Keep RF Kim S Hoff JT Edema form intracerebral hemorrhage: the role of thrombin. J Neurosurg (1996) 84:91–6. 10.3171/jns.1996.84.1.0091 8613842

[20] Wagner KR Xi G Hua Y Kleinholz M de Courten-Myers GM Myers RE Lobar intracerebral hemorrhage model in pigs: rapid edema development in perihematomal white matter. Stroke (1996) 27(3):490–7. 10.1161/01.STR.27.3.490 8610319

[21] Rosenberg GA Navratil M. Metalloproteinase inhibition blocks edema in intracerebral hemorrhage in the rat. Neurology (1997) 48(4):921–6. 10.1212/WNL.48.4.921 9109878

[22] Chen ST Chen SD Hsu CY Hogan EL. Progression of hypertensive intracerebral hemorrhage. Neurology (1989) 39(11):1509–14. 10.1212/WNL.39.11.1509 2812332

[23] Broderick JP Brott TG Tomsick T Barsan W Spilker J. Ultra-early evaluation of intracerebral hemorrhage. J Neurosurg (1990) 72(2):195–9. 10.3171/jns.1990.72.2.0195 2295917

[24] Fujitsu K Muramoto M Ikeda Y Inada Y Kim I Kuwabara T. Indications for surgical treatment of putaminal hemorrhage. Comparative study based on serial CT and time-course analysis. J Neurosurg (1990) 73(4):518–25. 10.3171/jns.1990.73.4.0518 2398381

[25] Kazui S Naritomi H Yamamoto H Sawada T Yamaguchi T. Enlargement of spontaneous intracerebral hemorrhage. Incidence and time course. Stroke (1996) 27(10):1783–7. 10.1161/01.STR.27.10.1783 8841330

[26] Brott T Broderick J Kothari R Barsan W Tomsick T Sauerbeck L Early hemorrhage growth in patients with intracerebral hemorrhage. Stroke (1997) 28(1):1–5. 10.1161/01.STR.28.1.1 8996478

[27] Dowlatshahi D Brouwers HB Demchuk AM Hill MD Aviv RI Ufholz LA Predicting intracerebral hemorrhage growth with the spot sign: the effect of onset-to-scan time. Stroke (2016) 47(3):695–700. 10.1161/STROKEAHA.115.012012 26846857 PMC4766058

[28] Fujii Y Takeuchi S Sasaki O Minakawa T Tanaka R. Multivariate analysis of predictors of hematoma enlargement in spontaneous intracerebral hemorrhage. Stroke (1998) 29(6):1160–6. 10.1161/01.STR.29.6.1160 9626289

[29] Qureshi AI Ezzeddine MA Nasar A Suri MF Kirmani JF Hussein HM Prevalence of elevated blood pressure in 563,704 adult patients with stroke presenting to the ED in the United States. Am J Emerg Med (2007) 25(1):32–8. 10.1016/j.ajem.2006.07.008 17157679 PMC2443694

[30] Mayer SA Kurtz P Wyman A Sung GY Multz AS Varon J Clinical practices, complications, and mortality in neurological patients with acute severe hypertension: the studying the treatment of acute hypertension registry. Crit Care Med (2011) 39(10):2330–6. 10.1097/CCM.0b013e3182227238 21666448

[31] Fischer U Cooney MT Bull LM Silver LE Chalmers J Anderson CS Acute post-stroke blood pressure relative to premorbid levels in intracerebral haemorrhage versus major ischaemic stroke: a population-based study. Lancet Neurol (2014) 13(4):374–84. 10.1016/S1474-4422(14)70031-6 24582530 PMC4238109

[32] Rose JC Mayer SA. Optimizing blood pressure in neurological emergencies. Neurocrit Care (2004) 1(3):287–99. 10.1385/NCC:1:3:287 16174926

[33] Dandapani BK Suzuki S Kelley RE Reyes-Iglesias Y Duncan RC. Relation between blood pressure and outcome in intracerebral hemorrhage. Stroke (1995) 26(1):21–4. 10.1161/01.STR.26.1.21 7839391

[34] Fogelholm R Avikainen S Murros K. Prognostic value and determinants of first-day mean arterial pressure in spontaneous supratentorial intracerebral hemorrhage. Stroke (1997) 28(7):1396–400. 10.1161/01.STR.28.7.1396 9227690

[35] Terayama Y Tanahashi N Fukuuchi Y Gotoh F. Prognostic value of admission blood pressure in patients with intracerebral hemorrhage. Keio Cooperative Stroke Study. Stroke (1997) 28(6):1185–8. 10.1161/01.STR.28.6.1185 9183348

[36] Hemphill JC III Bonovich DC Besmertis L Manley GT Johnston SC. The ICH score: a simple, reliable grading scale for intracerebral hemorrhage. Stroke (2001) 32(4):891–7. 10.1161/01.STR.32.4.891 11283388

[37] Willmot M Leonardi-Bee J Bath PM. High blood pressure in acute stroke and subsequent outcome: a systematic review. Hypertension (2004) 43(1):18–24. 10.1161/01.HYP.0000105052.65787.35 14662649

[38] Zhang Y Reilly KH Tong W Xu T Chen J Bazzano LA Blood pressure and clinical outcome among patients with acute stroke in Inner Mongolia, China. J Hypertens (2008) 26(7):1446–52. 10.1097/HJH.0b013e328300a24a 18551022

[39] Kazui S Minematsu K Yamamoto H Sawada T Yamaguchi T. Predisposing factors to enlargement of spontaneous intracerebral hematoma. Stroke (1997) 28(12):2370–5. 10.1161/01.STR.28.12.2370 9412616

[40] Ohwaki K Yano E Nagashima H Hirata M Nakagomi T Tamura A. Blood pressure management in acute intracerebral hemorrhage: relationship between elevated blood pressure and hematoma enlargement. Stroke (2004) 35(6):1364–7. 10.1161/01.STR.0000128795.38283.4b 15118169

[41] Rosand J Eskey C Chang Y Gonzalez RG Greenberg SM Koroshetz WJ. Dynamic single-section CT demonstrates reduced cerebral blood flow in acute intracerebral hemorrhage. Cerebrovasc Dis (2002) 14(3–4):214–20. 10.1159/000065681 12403954

[42] Siddique MS Fernandes HM Wooldridge TD Fenwick JD Slomka P Mendelow AD. Reversible ischemia around intracerebral hemorrhage: a single-photon emission computerized tomography study. J Neurosurg (2002) 96(4):736–41. 10.3171/jns.2002.96.4.0736 11990815

[43] Broderick JP Diringer MN Hill MD Brun NC Mayer SA Steiner T Determinants of intracerebral hemorrhage growth: an exploratory analysis. Stroke (2007) 38(3):1072–5. 10.1161/01.STR.0000258078.35316.30 17290026

[44] Garg RK Liebling SM Maas MB Nemeth AJ Russell EJ Naidech AM. Blood pressure reduction, decreased diffusion on MRI, and outcomes after intracerebral hemorrhage. Stroke (2012) 43(1):67–71. 10.1161/STROKEAHA.111.629493 21980211 PMC3246540

[45] Prabhakaran S Gupta R Ouyang B John S Temes RE Mohammad Y Acute brain infarcts after spontaneous intracerebral hemorrhage: a diffusion-weighted imaging study. Stroke (2010) 41(1):89–94. 10.1161/STROKEAHA.109.566257 19892994

[46] Powers WJ Zazulia AR Videen TO Adams RE Yundt KD Aiyagari V Autoregulation of cerebral blood flow surrounding acute (6 to 22 hours) intracerebral hemorrhage. Neurology (2001) 57(1):18–24. 10.1212/WNL.57.1.18 11445622

[47] Powers WJ Adams RE Yundt KD Acute pharmacological hypotension after intracerebral hemorrhage does not change cerebral blood flow. Stroke (1999) 30:242.

[48] Qureshi AI Wilson DA Hanley DF Traystman RJ Pharmacologic reduction of mean arterial pressure does not adversely affect regional cerebral blood flow and intracranial pressure in experimental intracerebral hemorrhage. Crit Care Med (1999) 27(5):965–71. 10.1097/00003246-199905000-00036 10362421

[49] Butcher K The Intracerebral Hemorrhage Acutely Decreasing Arterial Pressure Trial II (ICH-ADAPT II). (2016). Available from: https://clinicaltrials.gov/ct2/show/NCT02281838?term=adapt+ii&rank=1 10.1186/s12883-017-0884-4 PMC5437568 28525977

[50] Anderson CS Huang Y Wang JG Arima H Neal B Peng B Intensive blood pressure reduction in acute cerebral haemorrhage trial (INTERACT): a randomised pilot trial. Lancet Neurol (2008) 7(5):391–9. 10.1016/S1474-4422(08)70069-3 18396107

[51] Koch S Romano JG Forteza AM Otero CM Rabinstein AA. Rapid blood pressure reduction in acute intracerebral hemorrhage: feasibility and safety. Neurocrit Care (2008) 8(3):316–21. 10.1007/s12028-008-9085-8 18360781

[52] Qureshi AI Palesch YY Martin R Novitzke J Cruz-Flores S Ehtisham A Effect of systolic blood pressure reduction on hematoma expansion, perihematomal edema, and 3-month outcome among patients with intracerebral hemorrhage: results from the antihypertensive treatment of acute cerebral hemorrhage study. Arch Neurol (2010) 67(5):570–6. 10.1001/archneurol.2010.61 20457956 PMC5562043

[53] Qureshi AI Palesch YY. Antihypertensive treatment of acute cerebral hemorrhage (ATACH) II: design, methods, and rationale. Neurocrit Care (2011) 15(3):559–76. 10.1007/s12028-011-9538-3 21626077 PMC3340125

[54] Anderson CS Heeley E Huang Y Wang J Stapf C Delcourt C Rapid blood-pressure lowering in patients with acute intracerebral hemorrhage. N Engl J Med (2013) 368(25):2355–65. 10.1056/NEJMoa1214609 23713578

[55] Butcher KS Jeerakathil T Hill M Demchuk AM Dowlatshahi D Coutts SB The intracerebral hemorrhage acutely decreasing arterial pressure trial. Stroke (2013) 44(3):620–6. 10.1161/STROKEAHA.111.000188 23391776

[56] Anderson CS Huang Y Arima H Heeley E Skulina C Parsons MW Effects of early intensive blood pressure-lowering treatment on the growth of hematoma and perihematomal edema in acute intracerebral hemorrhage the intensive blood pressure reduction in acute cerebral haemorrhage trial (INTERACT). Stroke (2010) 41(2):307–12. 10.1161/STROKEAHA.109.561795 20044534

[57] Anderson CS Chalmers J Stapf C Blood-pressure lowering in acute intracerebral hemorrhage. N Engl J Med (2013) 369(13):1274–5. 10.1056/NEJMc1309586 24066751

[58] Qureshi AI. Antihypertensive treatment of acute cerebral hemorrhage (ATACH): rationale and design. Neurocrit Care (2007) 6(1):56–66. 10.1385/NCC:6:1:56 17356194

[59] Rincon F Mayer S. Novel therapies for intracerebral hemorrhage. Curr Opin Crit Care (2004) 10:94–100. 10.1097/00075198-200404000-00003 15075717

[60] Qureshi AI Palesch YY Barsan WG Hanley DF Hsu CY Martin RL Intensive blood-pressure lowering in patients with acute cerebral hemorrhage. N Engl J Med (2016) 375:1033–43. 10.1056/NEJMoa1603460 27276234 PMC5345109

[61] Hemphill JC Greenberg SM Anderson CS Becker K Bendok BR Cushman M Guidelines on the management of spontaneous intracerebral hemorrhage. Stroke (2015) 46:2032–60. 10.1161/STR.0000000000000069 26022637

[62] Mayer SA Brun NC Begtrup K Broderick J Davis S Diringer MN Recombinant activated factor VII for acute intracerebral hemorrhage. N Engl J Med (2005) 352(8):777–85. 10.1056/NEJMoa042991 15728810

[63] Mayer SA Brun NC Begtrup K Broderick J Davis S Diringer MN Efficacy and safety of recombinant activated factor VII for acute intracerebral hemorrhage. N Engl J Med (2008) 358(20):2127–37. 10.1056/NEJMoa0707534 18480205

[64] Mayer SA Davis SM Skolnick BE Brun NC Begtrup K Broderick JP Can a subset of intracerebral hemorrhage patients benefit from hemostatic therapy with recombinant activated factor VII? Stroke (2009) 40(3):833–40. 10.1161/STROKEAHA.108.524470 19150875

[65] Piriyawat P Morgenstern LB Yawn DH Hall CE Grotta JC Treatment of acute intracerebral hemorrhage with epsilon aminocaproic acid: a pilot study. Neurocrit Care (2004) 1(1):47–51. 10.1385/NCC:1:1:47 16174897

[66] Reza B Management of Intracerebral Hemorrhage with Aminocaproic Acid – Pilot Study (MANICHAN-PILOT). (2016). Available from: https://clinicaltrials.gov/ct2/show/NCT02639819

[67] Zazulia AR Antifibrinolytic Therapy in Acute Intracerebral Hemorrhage “ATICH”. (2016). Available from: http://www.strokecenter.org/trials/clinicalstudies/antifibrinolytic-therapy-in-acute-intracerebral-hemorrhage/description

[68] Flaherty ML STOP-IT. The Spot Sign for Predicting and Treating ICH Growth Study. (2012). Available from: http://www.stopitstudy.org/index.html

[69] Gladstone DJ “Spot Sign” Selection of Intracerebral Hemorrhage to Guide Hemostatic Therapy (SPOTLIGHT). (2011). Available from: https://clinicaltrials.gov/ct2/show/NCT01359202

[70] Liping L Tranexamic Acid for Acute ICH Growth Predicted by Spot Sign (TRAIGE). (2016). Available from: https://clinicaltrials.gov/ct2/show/NCT02625948

[71] Meretoja A STOP-AUST: The Spot Sign and Tranexamic Acid On Preventing ICH Growth – AUStralasia Trial (STOP-AUST). (2016). Available from: https://clinicaltrials.gov/ct2/show/NCT01702636 10.1111/ijs.12132 23981692

[72] Goldstein JN Fazen LE Snider R Schwab K Greenberg SM Smith EE Contrast extravasation on CT angiography predicts hematoma expansion in intracerebral hemorrhage. Neurology (2007) 68(12):889–94. 10.1212/01.wnl.0000257087.22852.21 17372123

[73] de Gans K de Haan RJ Majoie CB Koopman MM Brand A Dijkgraaf MG Patch: platelet transfusion in cerebral haemorrhage: study protocol for a multicentre, randomised, controlled trial. BMC Neurol (2010) 10:19. 10.1186/1471-2377-10-19 20298539 PMC2851678

[74] Steiner T Freiberger A Griebe M Husing J Ivandic B Kollmar R International normalised ratio normalisation in patients with coumarin-related intracranial haemorrhages – the INCH trial: a randomised controlled multicentre trial to compare safety and preliminary efficacy of fresh frozen plasma and prothrombin complex – study design and protocol. Int J Stroke (2011) 6(3):271–7. 10.1111/j.1747-4949.2010.00560.x 21557816

[75] Naidech AM Liebling SM Rosenberg NF Lindholm PF Bernstein RA Batjer HH Early platelet transfusion improves platelet activity and may improve outcomes after intracerebral hemorrhage. Neurocrit Care (2012) 16(1):82–7. 10.1007/s12028-011-9619-3 21837536 PMC6211165

[76] Naidech AM Maas MB Levasseur-Franklin KE Liotta EM Guth JC Berman M Desmopressin improves platelet activity in acute intracerebral hemorrhage. Stroke (2014) 45(8):2451–3. 10.1161/STROKEAHA.114.006061 25005444 PMC6211162

[77] Imberti R Pietrobono L Klersy C Gamba G Iotti G Cornara G. Intraoperative intravenous administration of rFVIIa and hematoma volume after early surgery for spontaneous intracerebral hemorrhage: a randomized prospective phase II study. Minerva Anestesiol (2012) 78(2):168–75. 21750485

[78] Thiex R Tsirka SE. Brain edema after intracerebral hemorrhage: mechanisms, treatment options, management strategies and operative indications. Neurosurg Focus (2007) 22:1–7. 10.3171/foc.2007.22.5.7 17613237

[79] Ziai W Hematology and inflammatory signaling of intracerebral hemorrhage. Stroke (2013) 44:S74–8. 10.1161/STROKEAHA.111.000662 23709738 PMC12054399

[80] Xi G Wagner KR Keep RF Hua Y de Courten-Myers GM Broderick JP The role of blood clot formation on early edema development after experimental intracerebral hemorrhage. Stroke (1998) 29:2580–6. 10.1161/01.STR.29.12.2580 9836771

[81] Xi G Keep RF Hoff JT. Erythrocytes and delayed brain edema formation following intracerebral hemorrhage in rats. J Neurosurg (1998) 89:991–6. 10.3171/jns.1998.89.6.0991 9833826

[82] Nath FP Kelly PT Jenkins A Mendelow AD Graham DI Teasdale GM. Effects of experimental intracerebral hemorrhage on blood flow, capillary permeability, and histochemistry. J Neurosurg (1987) 66:555–62. 10.3171/jns.1987.66.4.0555 3559721

[83] Gebel JM Jauch EC Brott TG Khoury J Sauerbeck L Salisbury S Natural history of PHE in patients with hyperacute spontaneous intracerebral hemorrhage. Stroke (2002) 33:2631–5. 10.1161/01.STR.0000035284.12699.84 12411653

[84] Lee KR Betz AL Keep RF Chenevert TL Kim S Hoff JT. Intracerebral infusion of thrombin as a cause of brain edema. J Neurosurg (1995) 83:1045–50. 10.3171/jns.1995.83.6.1045 7490619

[85] Li N Worthmann H Heeren M Schuppner R Deb M Tryc AB Temporal pattern of cytotoxic edema in the perihematomal region after intracerebral hemorrhage: a serial magnetic resonance imaging study. Stroke (2013) 44(4):1144–6. 10.1161/STROKEAHA.111.000056 23391767

[86] Brunswick AS Hwang BY Appelboom G Hwang RY Piazza MA Connolly ES Jr Serum biomarkers of spontaneous intracerebral hemorrhage induced secondary brain injury. J Neurol Sci (2012) 321(1–2):1–10. 10.1016/j.jns.2012.06.008 22857988

[87] McDonald JW Shapiro SM Silverstein FS Johnston MV Role of glutamate receptor-mediated excitotoxicity in bilirubin induced brain injury in the Gunn rat model. Exp Neurol (1998) 150:21–9. 10.1006/exnr.1997.6762 9514835

[88] Dziedzic T Bartus S Klimkowicz A Motyl M Slowik A Szczudlik A. Intracerebral hemorrhage triggers interleukin-6 and interleukin-10 release in blood. Stroke (2002) 33:2334–5. 10.1161/01.STR.0000027211.73567.FA 12215608

[89] Dinkel K Dhabhar FS Sapolsky RM. Neurotoxic effects of polymorphonuclear granulocytes on hippocampal primary cultures. Proc Natl Acad Sci U S A (2004) 101:331–6. 10.1073/pnas.0303510101 14684829 PMC314185

[90] Qureshi AI Ali Z Suri MF Shuaib A Baker G Todd K Extracellular glutamate and other amino acids in experimental intracerebral hemorrhage: an in vivo microdialysis study. Crit Care Med (2003) 31:1482–9. 10.1097/01.CCM.0000063047.63862.99 12771622

[91] Qureshi AI Suri MF Ostrow PT Kim SH Ali Z Shatla AA Apoptosis as a form of cell death in intracerebral hemorrhage. Neurosurgery (2003) 52:1041–8. 10.1227/01.NEU.0000057694.96978.BC 12699545

[92] Lee KR NMDA antagonists in ischemic stroke. Neurology (1997) 49:S66–9. 10.1212/WNL.49.5_Suppl_4.S66 9371155

[93] Lipton SA Rosenberg PA Excitatory amino acids as a final common pathway for neurologic disorders. N Engl J Med (1994) 330:613–22. 10.1056/NEJM199403033300907 7905600

[94] Castillo J Dávalos A Alvarez-Sabín J Pumar JM Leira R Silva Y Molecular signatures of brain injury after intracerebral hemorrhage. Neurology (2002) 58:624–9. 10.1212/WNL.58.4.624 11865143

[95] Megyeri P Abrahám CS Temesvári P Kovács J Vas T Speer CP Recombinant human tumor necrosis factor alpha constricts pial arterioles and increases blood-brain barrier permeability in newborn piglets. Neurosci Lett (1992) 148:137–40. 10.1016/0304-3940(92)90823-P 1300486

[96] Larrick JW Wright SC Cytotoxic mechanism of tumor necrosis factor alpha. FASEB J (1990) 4:3215–23. 2172061 10.1096/fasebj.4.14.2172061

[97] Wasserman JK Zhu X Schlichter LC. Evolution of the inflammatory response in the brain following intracerebral hemorrhage and effects of delayed minocycline treatment. Brain Res (2007) 1180:140–54. 10.1016/j.brainres.2007.08.058 17919462

[98] Del Bigio MR Yan HJ Buist R Peeling J. Experimental intracerebral hemorrhage in rats. Magnetic resonance imaging and histopathological correlates. Stroke (1996) 27(12):discussion 2319–20. 10.1161/01.STR.27.12.2312 8969799

[99] Barone FC Feuerstein GZ. Inflammatory mediators and stroke: new opportunities for novel therapeutics. J Cereb Blood Flow Metab (1999) 19(8):819–34. 10.1097/00004647-199908000-00001 10458589

[100] Xue M Del Bigio MR. Intracerebral injection of autologous whole blood in rats: time course of inflammation and cell death. Neurosci Lett (2000) 283(3):230–2. 10.1016/S0304-3940(00)00971-X 10754230

[101] Mayne M Ni W Yan HJ Xue M Johnston JB Del Bigio MR Antisense oligodeoxynucleotide inhibition of tumor necrosis factor-alpha expression is neuroprotective after intracerebral hemorrhage. Stroke (2001) 32(1):240–8. 10.1161/01.STR.32.1.240 11136943

[102] Peeling J Yan HJ Corbett D Xue M Del Bigio MR. Effect of FK-506 on inflammation and behavioral outcome following intracerebral hemorrhage in rat. Exp Neurol (2001) 167(2):341–7. 10.1006/exnr.2000.7564 11161622

[103] Wang J Tsirka SE. Contribution of extracellular proteolysis and microglia to intracerebral hemorrhage. Neurocrit Care (2005) 3(1):77–85. 10.1385/NCC:3:1:077 16159103

[104] Wang J Dore S. Inflammation after intracerebral hemorrhage. J Cereb Blood Flow Metab (2007) 27(5):894–908. 10.1038/sj.jcbfm.9600403 17033693

[105] Hammond MD Taylor RA Mullen MT Ai Y Aguila HL Mack M CCR2+Ly6 chi inflammatory monocyte recruitment exacerbates acute disability following intracerebral hemorrhage. J Neurosci (2014) 34(11):3901–9. 10.1523/JNEUROSCI.4070-13.2014 24623768 PMC3951693

[106] Fuerstein GZ Wang X Barone FC The role of cytokines in neuropathy of stroke and neurotrauma. Neuroimmunomodulation (1998) 5:143–59. 10.1159/000026331 9730680

[107] Feuerstein GZ Liu T Barone FC. Cytokines, inflammation, and brain injury: role of tumor necrosis factor-alpha. Cerebrovasc Brain Metab Rev (1994) 6:341–60. 7880718

[108] Scholz M Cinatl J Schädel-Höpfner M Windolf J Neutrophils and the blood brain barrier dysfunction after trauma. Med Res Rev (2007) 27:401–16. 10.1002/med.20064 16758487

[109] Wasserman JK Schlichter LC. Minocycline protects the blood-brain barrier and reduces edema following intracerebral hemorrhage in the rat. Exp Neurol (2007) 207:227–37. 10.1016/j.expneurol.2007.06.025 17698063

[110] Power C Henry S Del Bigio MR Larsen PH Corbett D Imai Y Intracerebral hemorrhage induces macrophage activation and matrix metalloproteinases. Ann Neurol (2003) 53:731–42. 10.1002/ana.10553 12783419

[111] Hua Y Xi G Keep RF Hoff JT. Complement activation in the brain after experimental intracerebral hemorrhage. J Neurosurg (2000) 92:1016–22. 10.3171/jns.2000.92.6.1016 10839264

[112] Hansch GM Shin MI Complement attack phase. In: Rother K Till GO Hansch GM editors. The Complement System. Heidelberg: Springer-Verlag (1998). p. 115–45.

[113] Abilleira S Montaner J Molina CA Monasterio J Castillo J Alvarez-Sabín J. Matrix metalloproteinase-9 concentration after spontaneous intracerebral hemorrhage. J Neurosurg (2003) 99:65–70. 10.3171/jns.2003.99.1.0065 12854746

[114] Gasche Y Fujimura M Morita-Fujimura Y Copin JC Kawase M Massengale J Early appearance of activated matrix metalloproteinase-9 after focal cerebral ischemia in mice: a possible role in blood-brain barrier dysfunction. J Cereb Blood Flow Metab (1999) 19:1020–8. 10.1097/00004647-199909000-00010 10478654

[115] Leppert D Ford J Stabler G Grygar C Lienert C Huber S Matrix metalloproteinase-9 (gelatinase B) is selectively elevated in CSF during relapses and stable f=phases of multiple sclerosis. Brain (1998) 121:2327–34. 10.1093/brain/121.12.2327 9874483

[116] Nelson AR Fingleton B Rothenberg ML Matrisian LM. Matrix metalloproteinases: biological activity and clinical implications. J Clin Oncol (2000) 18:1135–49. 10.1200/JCO.2000.18.5.1135 10694567

[117] Xi G Keep RF Hoff JT Mechanisms of brain injury after intracerebral hemorrhage. Lancet Neurol (2006) 5:53–63. 10.1016/S1474-4422(05)70283-0 16361023

[118] Hua Y Keep RF Hoff JT Xi G Brain injury after intracerebral hemorrhage. The role of thrombin and iron. Stroke (2007) 38:759–62. 10.1161/01.STR.0000247868.97078.10 17261733

[119] Hugli TE Complement Factors and Inflammation: Effects of Thrombin on Components of c3 and c5. Chemistry and Biology of Thrombin. Michigan: Ann Arbor Science Publishers (1977). p. 345–60.

[120] Hua Y Wu J Keep RF Nakamura T Hoff JT Xi G. Tumor necrosis factor alpha increases in the brain following intracerebral hemorrhage and thrombin stimulation. Neurosurgery (2006) 58:542–50. 10.1227/01.NEU.0000197333.55473.AD 16528196

[121] Noorbakhsh F Vergnolle N Hollenberg MD Power C Proteinase-activated receptors in the nervous system. Nat Rev Neurosci (2003) 4:981–90. 10.1038/nrn1255 14682360

[122] Sarker KP Yamahata H Nakata M Arisato T Nakajima T Kitajima I Recombinant thrombomodulin inhibits thrombin-induced vascular endothelial growth factor production in neuronal cells. Haemostasis (1999) 29:343–52. 10844408 10.1159/000022522

[123] Vaughan PJ Pike CJ Cotman CW Cunningham DD. Thrombin receptor activation protects neurons and astrocytes from cell death produced by environmental insults. J Neurosci (1995) 15:5389–401. 7623161 10.1523/JNEUROSCI.15-07-05389.1995 PMC6577903

[124] Matsuoka H Hamada R Role of thrombin in CNS damage associated with intracerebral haemorrhage: opportunity for pharmacological intervention? CNS Drugs (2002) 16(8):509–16. 10.2165/00023210-200216080-00001 12096932

[125] Sansing LH Kaznatcheeva EA Perkins CJ Komaroff E Gutman FB Newman GC. Edema after intracerebral hemorrhage: correlations with coagulation parameters and treatment. J Neurosurg (2003) 98:985–92. 10.3171/jns.2003.98.5.0985 12744358

[126] Kitaoka T Hua Y Xi G Hoff JT Keep RF. Delayed argatroban treatment reduces edema in a rat model of intracerebral hemorrhage. Stroke (2002) 33:3012–8. 10.1161/01.STR.0000037673.17260.1B 12468805

[127] Hua Y Xi G Keep RF Wu J Jiang Y Hoff JT. Plasminogen activator inhibitor-1 induction after experimental intracerebral hemorrhage. J Cereb Blood Flow Metab (2002) 22:55–61. 10.1097/00004647-200201000-00007 11807394

[128] Pulsinelli WA Waldman S Rawlinson D Plum F. Moderate hyperglycemia augments ischemic brain damage: a neuropathologic study in the rat. Neurology (1982) 32:1239–46. 10.1212/WNL.32.11.1239 6890157

[129] Holmin S Mathiesen T Intracerebral administration of interleukin 1 beta and induction of inflammation, apoptosis, and vasogenic edema. J Neurosurg (2000) 92:108–20. 10.3171/jns.2000.92.1.0108 10616089

[130] Asakawa H Miyagawa J Hanafusa T Kuwajima M Matsuzawa Y High glucose and hyperosmolarity increase secretion of interleukin-1beta in cultured human aortic endothelial cells. J Diabetes Complications (1997) 11:176–9. 9174899 10.1016/s1056-8727(97)00004-4

[131] Pampfer S Cordi S Dutrieux C Vanderheyden I Marchand C De Hertogh R Interleukin 1 beta mediates the effect of high d-glucose on the section of TNF-alpha by mouse uterine epithelial cells. Cytokine (1999) 11:500–9. 10.1006/cyto.1998.0459 10419651

[132] Song EC Chu K Jeong SW Jung KH Kim SH Kim M Hyperglycemia exacerbates brain edema and perihematomal cell death after intracerebral hemorrhage. Stroke (2003) 34:2215–20. 10.1161/01.STR.0000088060.83709.2C 12907821

[133] Schulz J Plesnila N Eriskat J Stoffel M Pruneau D Baethmann A. LF16-0687 a novel non-peptide bradykinin B2 receptor antagonist reduces vasogenic brain edema from a focal lesion in rats. Acta Neurochir Suppl (2000) 76:137–9. 11449993 10.1007/978-3-7091-6346-7_28

[134] Park CC Shin ML Simard JM. The complement membrane attack complex and the bystander effect in cerebral vasospasm. J Meurosurg (1997) 87:294–333. 10.3171/jns.1997.87.2.0294 9254096

[135] Kase CS Caplan LR Intracerebral Hemorrhage. Boston, MA: Butterworth-Heinemann (1994).

[136] Walgren NG Lindquist C Heme derivatives in the cerebrospinal fluid after intracranial hemorrhage. Eur Neurol (1987) 26:216–21. 10.1159/000116339 3595660

[137] Gutteridge JMC The antioxidant activity of haptoglobin towards haemoglobin-stimulated lipid peroxidation. Biochim Biophys Acta (1987) 917(2):219–23. 10.1016/0005-2760(87)90125-1 2879568

[138] Matz P Turner C Weinstein PR Massa SM Panter SS Sharp FR. Heme-oxygenase-1 induction in glia throughout rat brain following experimental subarachnoid hemorrhage. Brain Res (1996) 713:211–22. 10.1016/0006-8993(95)01511-6 8724993

[139] Koeppen AH Dickson AC McEvoy JA. The cellular reactions to experimental intracerebral hemorrhage. J Neurol Sci (1995) 134:102–12. 10.1016/0022-510X(95)00215-N 8847540

[140] Willmore LJ Rubin JJ. Formation of malonaldehyde and focal brain edema induced by subpial injection of FeCl2 into rat isocortex. Brain Res (1982) 246:113–9. 10.1016/0006-8993(82)90147-0 7127081

[141] Huang FP Xi G Keep RF Hua Y Nemoianu A Hoff JT. Brain edema after experimental intracerebral hemorrhage: role of hemoglobin degradation products. J Neurosurg (2002) 96:287–93. 10.3171/jns.2002.96.2.0287 11838803

[142] Suttner DM Dennery PA. Reversal of HO-1 related cytoprotection with increased expression is due to reactive iron. FASEB J (1999) 13:1800–9. 10506583 10.1096/fasebj.13.13.1800

[143] Xi G Keep RF Hua Y Xiang J Hoff JT. Attenuation of thrombin-induced brain edema by cerebral thrombin preconditioning. Stroke (1999) 30:1247–55. 10.1161/01.STR.30.6.1247 10356108

[144] Nakamura T Keep RF Hua Y Schallert T Hoff JT Xi G Deferoxamine-induced attenuation of brain edema and neurological deficits in a rat model of intracerebral hemorrhage. J Neurosurg (2004) 100:672–8. 10.3171/jns.2004.100.4.0672 15070122

[145] Wu J Hua Y Keep RF Nakamura T Hoff JT Xi G. Iron and iron-handling proteins in the brain after intracerebral hemorrhage. Stroke (2003) 34:2964–9. 10.1161/01.STR.0000103140.52838.45 14615611

[146] Regan RF Yapin G Toxic effect of hemoglobin on spinal cord neurons in culture. J Neurotrauma (2009) 15(8):645–53. 10.1089/neu.1998.15.645 9726263

[147] Wu J Hua Y Keep RF Schallert T Hoff JT Xi G. Oxidative brain injury from extravasated erythrocytes after intracerebral hemorrhage. Brain Res (2002) 953:45–52. 10.1016/S0006-8993(02)03268-7 12384237

[148] Rincon F Friedman DP Bell R Mayer SA Bray PF Targeted temperature management after intracerebral hemorrhage (TTM-ICH): methodology of a prospective randomized clinical trial. Int J Stroke (2014) 9:646–51. 10.1111/ijs.12220 24450819

